# Effects of Intramammary Antimicrobial Treatment on the Milk Microbiota Composition in Mild Clinical Bovine Mastitis Caused by Gram-Positive Bacteria

**DOI:** 10.3390/ani13040713

**Published:** 2023-02-17

**Authors:** Mayu Hayashi, Yasunori Shinozuka, Tomomi Kurumisawa, Takuya Yagisawa, Nagomu Suenaga, Yuko Shimizu, Naoki Suzuki, Kazuhiro Kawai

**Affiliations:** 1 School of Veterinary Medicine, Azabu University, 1-17-71 Fuchinobe, Sagamihara 252-5201, Japan; 2 Mastitis Research Center, Azabu University, 1-17-71 Fuchinobe, Sagamihara 252-5201, Japan; 3 Hokkaido Agriculture Mutual Aid Association, 4-1-1, Sapporo 060-0004, Japan; 4 Graduate School of Integrated Sciences for Life, Hiroshima University, 1-4-4 Kagamiyama, Higashi-Hiroshima 739-8528, Japan

**Keywords:** 16S rRNA-based metagenomic analysis, gram-positive bacteria, intramammary antimicrobial treatment, mastitis, milk microbiota

## Abstract

**Simple Summary:**

Because bovine mastitis is usually a result of bacterial infection, treatment with antimicrobial agents is widely used, and mastitis is the main reason for the use of antimicrobial agents in dairy cows. Although it is generally understood that antimicrobial treatment affects various microbiota in the body, the effects of intramammary administration of antimicrobials for mastitis on the milk microbiota in cows remain unclear. This study is the first to examine the changes in the milk microbiota during intramammary administration of antimicrobials for mastitis caused by Gram-positive bacteria. We found that the effect of the antimicrobial drug treatment on the milk microbiota composition became evident after the second day of antimicrobial administration, and similarities in the milk microbiota composition among the antimicrobial treatment group were discovered on the seventh day. This is the first study to report the changes in the milk microbiota during intramammary antimicrobial treatment for mild clinical mastitis caused by Gram-positive bacteria in dairy cattle.

**Abstract:**

The purpose of this study was to clarify the effects of antimicrobial treatment for mild mastitis caused by Gram-positive bacteria on the milk microbiota in dairy cattle. Sixteen quarters of sixteen cows with mild clinical mastitis from the same herd were included in the study. On the day of onset (day 0), the cows were randomly allocated to a no-treatment (NT; *n* = 10) group or an intramammary antimicrobial treatment (AMT) group that received AMT starting on day 0 (AMT-AMT group; *n* = 6). The next day (day 1), the cows in the NT group were randomly allocated into an NT group (NT-NT group; *n* = 3) that received no treatment or an AMT group that received AMT starting on day 1 (NT-AMT group; *n* = 7). Milk samples were collected on days 0, 1, 3 and 7, and the milk microbiota of each sample was comprehensively analyzed via 16S rRNA gene amplicon sequencing of the milk DNA. During the treatment period, the milk microbiota of the NT-NT group did not change, but those of the NT-AMT and AMT-AMT groups changed significantly on days three and seven. Thus, the use of antimicrobials for mild mastitis caused by Gram-positive bacteria changes the milk microbiota composition.

## 1. Introduction

Bovine mastitis is the most common disease in dairy cows, with an incidence of approximately 28% in Japan [1]. Because the main cause of mastitis is bacterial infection, antimicrobial agents are commonly used to treat the condition. In fact, mastitis is the main reason for the use of antimicrobials in dairy cows. Mastitis without systemic symptoms (mild mastitis) accounts for approximately 85% of all mastitis cases, and it has been reported that 32% of cases are caused by Gram-positive bacteria, 30% of cases are culture-negative, 28% of cases are caused by Gram-negative bacteria, and 10% of cases are caused by other pathogens [2]. To treat mild mastitis, intramammary antimicrobials are generally administered for three days.

In recent years, the presence of mammary microbiota has received increasing attention and has been reported in both humans and cattle. It has been studied more extensively in humans than in cattle, and studies in humans have helped us to understand the microbiota in the quarter of cows, for which there is still little information. In humans, the milk microbiota is implicated in the colonization of infant intestines and affects the health of infants [3]. Similarly, many of the bacteria found in bovine colostrum are also found in the gastrointestinal tract of calves, contributing to the composition of their intestinal microbiota [4]. In humans, *Staphylococcus* spp., *Streptococcus* spp., *Lactobacillus* spp. and *Bifidobacterium* spp. have been reported to be dominant in the milk microbiota of healthy breasts [3], while *Clostridium* spp., members of the family *Lachnospiraceae*, members of the family *Ruminococcaceae*, *Bifidobacterium* spp. and *Bacteroides* spp. are abundant in the milk microbiota of healthy cows [5]. The milk microbiota of mastitic quarters of cows have low diversity; in particular, *Staphylococcus* spp. and *Corynebacterium* spp. dominate [5]. Additionally, although it has been reported that the diversity of the milk microbiota of non-mastitic cows was high, regardless of the somatic cell count (SCC), *Staphylococcus* and *Streptococcus* were common genera [6]. The microbiota in healthy human breasts and quarters of cattle mammary glands are highly diverse and are abundant in members of the phyla Firmicutes and Actinobacteria, whereas those in mastitic quarters are less diverse, and the reported abundances of bacteria present vary from study to study.

The effects of antimicrobial treatment on the gut microbiota, which are thought to affect the mammary microbiota, have been studied in humans and cattle. Previous studies have reported that the administration of antimicrobials to the gut reduces the diversity of the gut microbiota [7,8,9], but does not necessarily increase or decrease the number of bacteria belonging to a specific phylum [7,8,9,10] depending on the method used in the study.

The effects of antimicrobial treatment on the mammary microbiota have also been partially studied in humans and cattle. In humans, it has been reported that the abundance of *Lactobacillus* spp. and *Bifidobacterium* spp. was significantly lower in women treated with antimicrobials during pregnancy and lactation [11]. Additionally, antimicrobial exposure during delivery altered the abundance and diversity of bacteria in breast milk in the first month after delivery, i.e., members of the phyla Proteobacteria and Firmicutes, especially *Streptococcaceae* and *Staphylococcaceae*, were abundant [12,13]. In healthy dry cows, antimicrobial treatment did not alter the SCC or bacterial abundance and did not affect the mean relative abundance of mastitis-causing bacteria, such as *Staphylococcus* spp. and *Streptococcus* spp., *Escherichia* spp., *Klebsiella* spp., *Mycoplasma* spp. [14]. These reports indicate that the effects of antimicrobial treatment on the mammary microbiota differ between humans and cattle. Thus, the microbiota change in the milk of dairy cows caused by administration of antimicrobials should be clarified in addition to that of human milk. It has become clear from several reports that the administration of antimicrobials affects the gut microbiota at the phylum and family levels, but it is unlikely to cause significant changes to the mammary microbiota of healthy quarters; however, it remains unclear how the administration of antimicrobials affects the mammary microbiota of mastitic quarters. Clarification of this point will enable a better understanding of mastitis, including the possibility that mastitis may reflect a microbial imbalance in the mammary gland. Therefore, this study aimed to clarify the impact of antimicrobial treatment on the microbiota and the clinical course of mastitic mammary glands.

## 2. Materials and Methods

### 2.1. Animals and Experimental Design

The present study was performed at a commercial dairy farm in Hokkaido Prefecture, Japan. Of the cows that developed clinical mastitis, 16 quarters of 16 cows were diagnosed with mild mastitis by a clinical veterinarian based on the classification of Roberson et al. [15]. Mild mastitis was diagnosed based on the absence of systemic symptoms. The day of mastitis onset was defined as day 0; the day after onset was defined as day 1. The 16 cows were randomly divided into three groups: one group received no treatment (NT) on day 0 and 1 (the NT-NT group; *n* = 3); one group received NT on day 0 and started receiving intramammary antimicrobial treatment (AMT) on day 1 (the NT-AMT group; *n* = 7); the remaining group started receiving AMT on day 0 (the AMT-AMT group; *n* = 6). The antimicrobial substance used to treat the mastitis was a mixture of penicillin (300,000 unit) and kanamycin (300 mg), both of which are approved in Japan for intramammary injections, and it was administered by the dairy farmer in compliance with the dose and usage instructions provided by the veterinarians. AMT was administered for a period deemed necessary and sufficient by the clinical veterinarian until clinical signs disappeared; no antibiotic treatment was administered to the NT-NT group. Seven days after onset, the clinical veterinarian examined the cows to determine the presence (or lack of) healthy milk that could be shipped for sale. As the composition of the milk microbiota does not differ based on the sampling methods used, e.g., collection via hand squeezing, a teat canal cannula or trans-teat-wall needle aspiration [16], we decided to collect samples via hand squeezing because it is a non-invasive procedure from the viewpoint of animal welfare. On days 0, 1, 3 and 7, after milking was finished and wiping with an alcohol swab was complete, milk samples were aseptically collected into tubes by a trained farmer. A total of 66 samples were successfully collected during the experimental period. The samples were immediately stored at 4 °C for bacterial identification and frozen at −20 °C for DNA extraction, respectively, and transported to the laboratory as soon as possible after each sampling. The farm owner provided permission for the sampling and for the use of the data obtained in this study.

In this study, the clinical veterinarian judged whether the cows were clinically cured based on the target quarter having no clinical symptoms and on whether the milk from the target quarter could be shipped for sale. All cows in the three groups in this study were eventually clinically cured.

### 2.2. Bacterial Cultures and Sensitivity Tests

Bacterial cultures and antimicrobial sensitivity tests were performed using the 16 samples collected prior to treatment. We directly cultured ten microliters of each milk sample on a sheep-blood agar plate (Nissui Pharmaceutical Co., Ltd., Tokyo, Japan). After 24 to 48 h of aerobic incubation at 37 °C, the obtained colonies were subcultured to obtain pure cultures, and identified using BD BBL CRYSTAL GP (Becton, Dickinson and Co., Franklin Lakes, NJ, USA) based on the results of Gram staining.

The antimicrobial sensitivity of the bacteria isolated from each milk sample to penicillin and kanamycin was tested using the agar disk diffusion further simplified (simplified-ADD) method, as previously reported [17].

### 2.3. Milk Test

The SCC and the lysosomal N-acetyl-β-D-glucosaminidase (NAGase) activity values were determined for each milk sample collected on days 0, 1, 3 and 7. Based on the method of Kawai et al. [18], the SCC was calculated using the electronic cell counter DCC (DeLaval International AB, Tumba, Sweden). The obtained SCC was transformed to somatic cell score (SCS) using the method of Wiggans and Shook [19]. Briefly, the SCS were calculated from SCC by:

SCS = log2 (SCC/100) + 3

where SCC was in units of cells per microliter. The NAGase activity was calculated using the β-N-acetylglucosaminidase Assay Kit (Sigma-Aldrich Co., LLC, St. Louis, MO, USA). The method of production is as follows: The whey was obtained by centrifuging milk samples at 3000 rpm for 10 min at 20 °C for the determination of the NAGase activity. Next, the absorbance of the unreacted substrate in the whey sample was deducted as background control from the absorbance of the same whey sample to avoid the effect of the whey color, and the NAGase activity was calculated.

### 2.4. DNA Extraction, 16S rRNA Gene Amplicon Sequencing, and Preprocessing of Sequence Reads

Genomic DNA from each milk sample was isolated using ISOSPIN Fecal DNA (Nippon Gene, Tokyo, Japan) following the manufacturer’s directions. DNA was isolated using silica membrane spin columns from milk samples subjected to mechanical disruption via bead beating. By measuring both the 260/280 and 260/230 absorbance ratios on a NanoDrop (NanoDrop Technologies, Wilmington, DE, USA), the quantity and purity of the extracted DNA were calculated. The V1–V9 region of the 16S rRNA gene was amplified from genomic DNA by a polymerase chain reaction (PCR) condition described in Table 1. PCR amplification of the 16S rRNA gene was performed using KAPA HiFi HotStart ReadyMix (Nippon Genetics, Tokyo, Japan) with a total volume of 1 µL including the inner primer pairs (0.25 µM each). Next, the resulting PCR products were targeted to attach a pair of barcodes at both ends under the conditions of 2 min at 30 °C and 2 min at 80 °C using the Rapid Barcodes in the Sequencing Barcoding Kit (SQK-RBK110.96; Oxford Nanopore Technologies, Oxford, UK). All PCR products were purified using AMPure^®^ XP (Beckman Coulter, Brea, CA, USA). The purity of PCR products was determined according to the absorbance with NanoDrop described above and the quantity was detected using a Quantus^TM^ Fluorometer (Promega, Madison, WI, USA). The barcoded samples were pooled and attached to sequencing adapters (Rapid Adapter, Oxford Nanopore Technologies, Oxford, UK) supplied in the Sequencing Barcoding Kit under the conditions of 5 min at room temperature to make a DNA library. Sequencing with the MinION^TM^ Mk1C (Oxford Nanopore Technologies, Oxford, UK) was carried out after the prepared DNA library (12 µL) was mixed with 37.5 µL of sequencing buffer, 25.5 µL of loading beads and loaded into the Spot-on Flow Cell R9 version (FLO-MIN106D; Oxford Nanopore Technologies, Oxford, UK). Data obtainment and FASTQ files preparation was performed using MINKNOW software ver. 21.11.6 (Oxford Nanopore Technologies, Oxford, UK). The resulting FASTQ files were trimmed and filtered by Nanofilt [20] software with filtering set to a minimum average read quality score of less than 10, all sequences shorter than 500 nucleotides were removed and the first 50 nucleotides of all reads were trimmed. After trimming and size selection, on average, 24,896 reads per sample (maximum, 69,256; minimum, 937) passed and were retained for bacterial identification. For each read, a minimap2 search with 5850 representative bacterial genome sequences stored in the Genome Sync database [21] was performed. The taxa were determined based on the National Center for Biotechnology Information taxonomy database [22]. Low-abundance taxa (less than 0.01% of the total reads) were discarded from the analysis.

### 2.5. Statistical Analyses

For the three groups, i.e., the NT-NT, NT-AMT and AMT-AMT groups, statistical analyses were conducted for the SCS, NAGase activity values and milk microbiota. Because the SCS and NAGase activity values were normalized by the Kolmogorov–Smirnov test, the relationship between these parameters and each antimicrobial treatment condition was examined via repeated measures analysis of variance (ANOVA), which is a parametric test. Because the richness and alpha diversity metrics (Simpson’s index and Shannon–Wiener index) of milk microbiota were not normalized by the Kolmogorov–Smirnov test, the relationships between each of these parameters and the antimicrobial treatment conditions were examined using the Friedman test, which is a non-parametric test. A *p* value of <0.05 was considered indicative of a statistically significant difference. All statistical analyses were performed with EZR (ver. 1.55; Saitama Medical Center, Jichi Medical University, Saitama, Japan), which is for R (The R Foundation for Statistical Computing, Vienna, Austria) [24]. More precisely, it is a modified version of the R commander designed to add statistical functions frequently used in biostatistics. Principal coordinate analysis (PCoA) two-dimensional visualization of the multidimensional milk microbiota similarities using microbiome distances and the analysis of similarities (ANOSIM) test were used as beta diversity metrics to evaluate the differences among days 0, 1, 3 and 7 for each treatment group based on the Bray–Curtis distance measure [25]. Furthermore, a comparison among the three groups on each day was performed in the same way. These data were analyzed using the PAST 4.09 software package [26]. The data for genus clusters were obtained, and bacterial taxa that were significantly enriched in a certain sample group were extracted via linear discriminant analysis effect size (LEfSe) analysis using the online Galaxy interface [27]. For LEfSe analysis, the alpha value for the factorial Kruskal–Wallis test was set to <0.05, and the threshold logarithmic linear discriminant analysis score for discriminative features was set to <2.0.

## 3. Results

### 3.1. Cow Characteristics

Grouping methods based on the antimicrobial use for each treatment group and each characteristic are described in Table 2. There were no significant differences in background factors among treatment groups. Although we did not know their medical history, we ensured that the cows enrolled in this study were clinically healthy prior to mastitis. All cows’ mastitic quarters were diagnosed as clinically cured by a veterinarian after seven days and shipping milk resumed.

### 3.2. Bacterial Isolation and Antimicrobial Sensitivity Tests

The bacterial cultures revealed that the mastitis-causing bacteria were coagulase-negative staphylococci and *Streptococcus* spp. other than *Streptococcus uberis*. The isolated bacteria from the 16 samples and the results from the disk diffusion method using the simplified ADD are shown in Table 3. All samples showed sensitivity to penicillin and/or kanamycin.

### 3.3. Milk SCS and NAGase Activity

**Figure 1:**
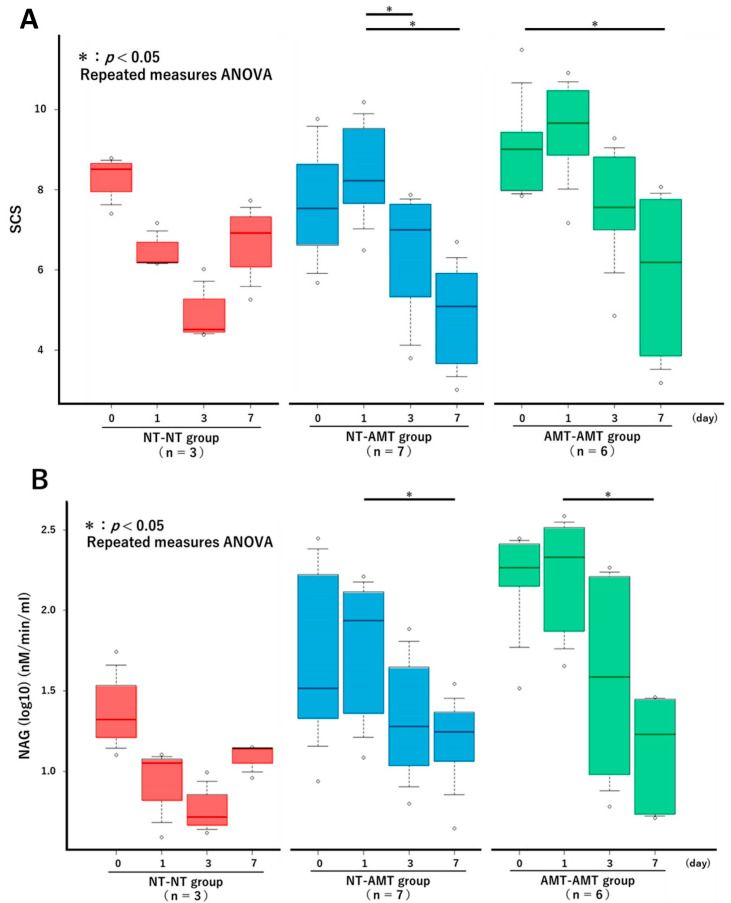
The changes in the Somatic Cell Score (**A**) and NAGase activity (**B**) over time (day 0, 1, 3, 7) in each treatment group (NT-NT, NT-AMT, AMT-AMT) for mastitis with Gram-positive bacteria. Bars represent standard deviation of the mean. Asterisks represent significant differences from the results of the repeated measures ANOVA among the days within the same group. (**A**) The changes in the SCS over time in each treatment group. There were no significant changes in the NT-NT group, but there were significant reductions between days 1 and 3, and days 1 and 7 in the NT-AMT group, and between days 0 and 7 in the AMT-AMT group. (**B**) The changes in the NAGase activity over time in each treatment group. There were no significant changes in the NT-NT group, but there were significant reductions between days 1 and 7 in the NT-AMT and AMT-AMT groups.

The changes in the SCS over time in each treatment group from the repeated measures ANOVA are shown in Figure 1A. There were no significant changes in the NT-NT group, but there were significant reductions in the NT-AMT and AMT-AMT groups. In particular, significant reductions were seen between days 1 and 3 and days 1 and 7 in the NT-AMT group and between days 0 and 7 in the AMT-AMT group. The changes in the NAGase activity over time in each treatment group are shown in Figure 1B. There were no significant changes in the NT-NT group, but there were significant reductions in the NT-AMT and AMT-AMT groups. In particular, significant reductions were noted between days 1 and 7 in both the NT-AMT and AMT-AMT groups.

### 3.4. Microbial Diversity

**Figure 2:**
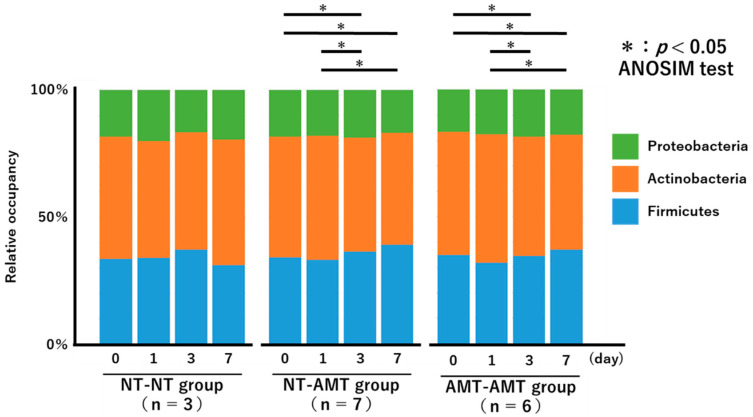
A 100% stacked bar chart showing the relative proportions of the phyla of the identified bacteria over time (day 0, 1, 3, 7) in each treatment group (NT-NT, NT-AMT, AMT-AMT) for mastitis with Gram-positive bacteria. Asterisks represent significant differences from the results of the ANOSIM test among the days within the same group. The three most abundant phyla are Actinobacteria, Proteobacteria and Firmicutes. According to the results of the ANOSIM test, there was no significant change in the NT-NT group, but significant changes were seen between days 0 and 3, days 0 and 7, days 1 and 3 and days 1 and 7 in the NT-AMT group, and between days 0 and 3, days 0 and 7, days 1 and 3 and days 1 and 7 in the AMT-AMT group.

The relative proportions of the phyla of the isolated bacteria from day 0 to day 7 for each treatment group are shown in Figure 2 as a 100% stacked bar chart. The figure shows only the three most abundant phyla, i.e., Actinobacteria, Proteobacteria, and Firmicutes, although members of Tenericutes and Bacteroidetes were also present in very small numbers.

**Figure 3:**
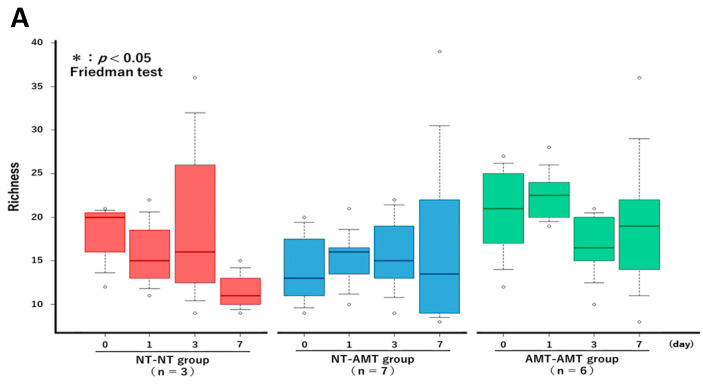

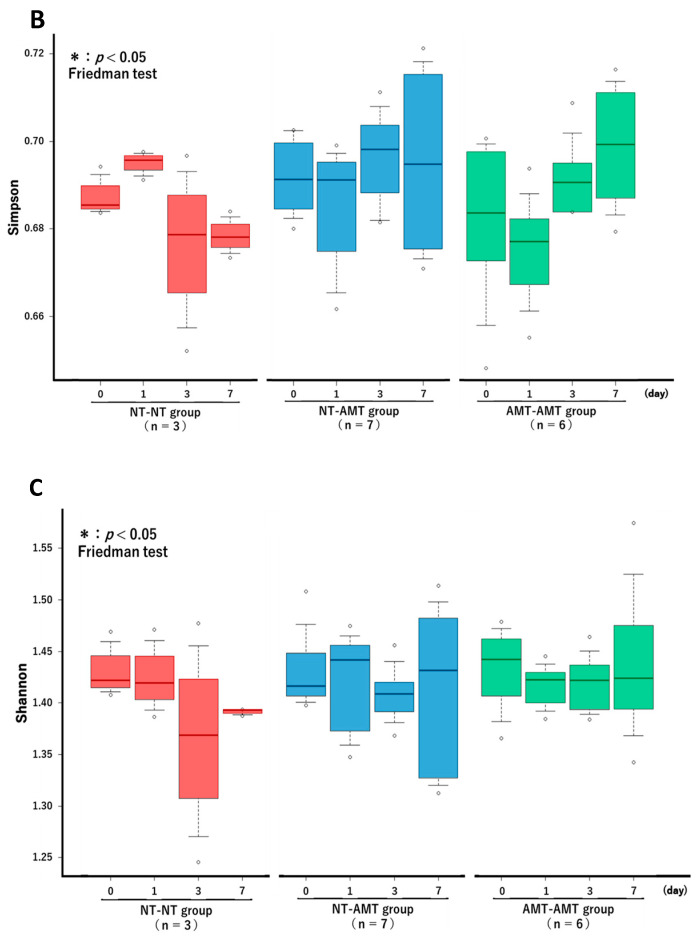
The changes in the Richness (**A**), Simpson’s index (**B**) and Shannon–Wiener index (**C**) over time (day 0, 1, 3, 7) in each treatment group (NT-NT, NT-AMT, AMT-AMT) for mastitis with Gram-positive bacteria. Bars represent standard deviation of the mean. Day 0 to day 7 changes in the richness and alpha diversity in each treatment group, as analyzed by the Friedman test. There were no significant differences among days (day 0, 1, 3, 7) within each treatment group in either Richness (**A**), Simpson’s index (**B**) or Shannon–Wiener index (**C**).

The Friedman test showed that there were no significant changes in the richness (Figure 3A) and alpha diversity metrics (Simpson’s index (Figure 3B) and Shannon–Wiener index (Figure 3C)) of the milk microbiota in all treatment groups. However, the ANOSIM test showed that in the NT-AMT group, there were significant changes between days 0 and 3, days 0 and 7, days 1 and 3 and days 1 and 7, whereas in the AMT-AMT group, there were significant changes between days 0 and 3, days 0 and 7, days 1 and 3 and days 1 and 7; only the NT-NT group, which was not treated with antimicrobials, showed no significant changes (Figure 2).

**Figure 4:**
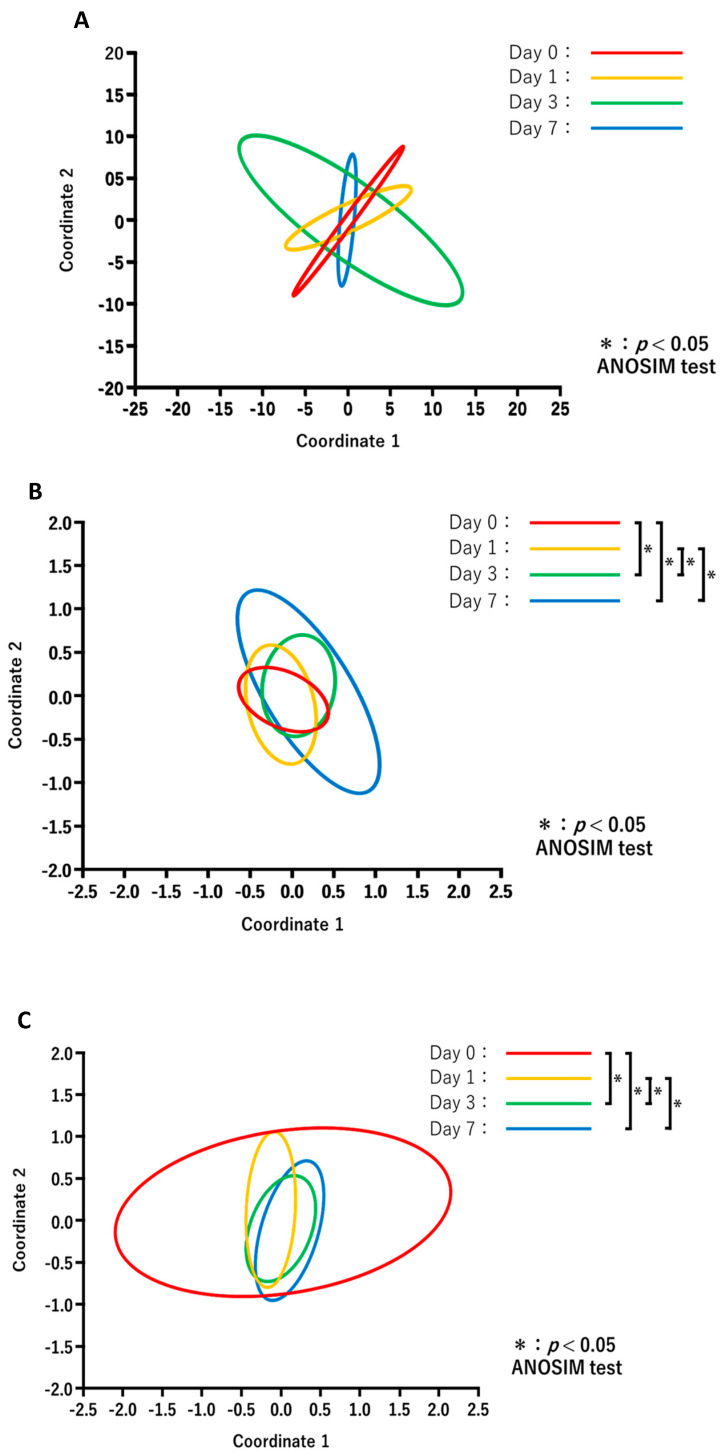
Principal coordinate analysis and ANOSIM test based on the Bray–Curtis distances and comparing the microbiome data of day 0, 1, 3 and 7 in each treatment of NT-NT group (**A**), NT-AMT group (**B**) and AMT-AMT group (**C**). (**A**) Comparison of the diversity between the days after treatment in the NT-NT group. There were no significant differences between the days after treatment. (**B**) Comparison of the diversity between the days after treatment in the NT-AMT group. There were significant differences between days 0 and 3, days 0 and 7, days 1 and 3 and days 1 and 7. (**C**) Comparison of the diversity between the days after treatment in the AMT-AMT group. There was a significant difference between days 0 and 3, days 0 and 7, days 1 and 3 and days 1 and 7.

The results of PCoA in each treatment group are shown in Figure 4. Comparisons of the diversity between the days after treatment for each group revealed no significant changes in the NT-NT group (Figure 4A), whereas the microbiota composition in the NT-AMT (Figure 4B) and AMT-AMT (Figure 4C) groups changed significantly on day 3 and day 7.

**Figure 5:**
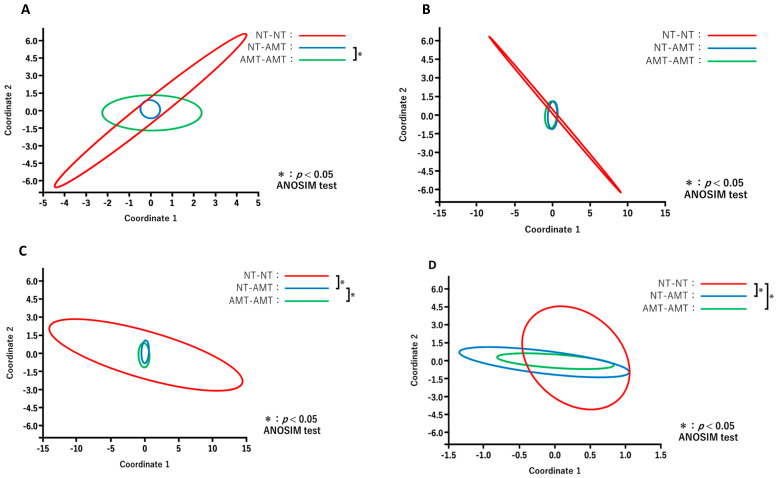
Principal coordinate analysis and ANOSIM test based on the Bray–Curtis distance comparing the microbiome data on day 0 (**A**), day 1 (**B**), day 3 (**C**) and day 7 (**D**) after treatment in each treatment group (NT-NT group, NT-AMT group and AMT-AMT group). (**A**) Comparison of the diversity between the treatment groups on day 0 after treatment. There was a significant difference between the NT-AMT and AMT-AMT groups. (**B**) Comparison of the diversity between treatment groups on day 1 after treatment. There were no significant differences between the groups. (**C**) Comparison of the diversity between the treatment groups on day 3 after treatment. There were significant differences between the NT-NT and NT-AMT groups, and the NT-AMT and AMT-AMT groups. (**D**) Comparison of the diversity between the treatment groups on day 7 after treatment. There were significant differences between the NT-NT and NT-AMT groups, and the NT-NT and AMT-AMT groups.

The results of PCoA for different days after treatment between the treatment groups are shown in Figure 5. Comparisons of the diversity among three groups on each day revealed that the constituent bacterial species of milk microbiota in the antimicrobial group, such as NT-AMT and AMT-AMT groups, differed on day 0 (Figure 5A) and day 3 (Figure 5C), but were similar on day 7 (Figure 5D).

**Figure 6:**
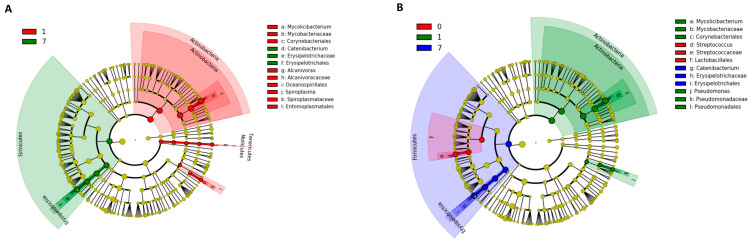
Variation in bacterial species in milk microbiota during 0–7 days on NT-AMT group (**A**) and AMT-AMT group (**B**) treated with antibiotics for mastitis with Gram-positive bacteria. Bacterial species that changed in abundance from the results of the Kruskal–Wallis test using LEfSe analysis. (**A**) Bacterial species that changed in abundance between the days after treatment in the NT-AMT group. There were many Actinobacteria on day 1 and many Firmicutes on day 7. (**B**) Bacterial species that changed in abundance between the days after treatment in the AMT-AMT group. There were many Actinobacteria on day 1 and many Firmicutes on day 7.

The results of the Kruskal–Wallis test using LEfSe analysis are shown in Figure 6. At the phylum level in the NT-AMT and AMT-AMT groups, the relative abundance of Actinobacteria was high on day 1, and that of Firmicutes was high on day 7 (Figure 6). In both the NT-AMT and AMT-AMT groups, the most common families were Mycobacteriaceae among the Actinobacteria on day 1 and Erysipelotrichaceae among the Firmicutes on day 3 and day 7.

## 4. Discussion

In the AMT group—such as, for example, in the NT-AMT and AMT-AMT groups—there was no change in the composition of the milk microbiota the day after starting treatment, but changes could be detected on days 3 and 7, and the fluctuating species from day 1 to days 3 and 7 were similar for the NT-AMT and AMT-AMT groups. Although the sensitivity of the mastitis-causing bacteria to antimicrobial agents was tested, sensitivity testing was not performed for the other bacteria that make up the milk microbiota. Therefore, the effects of antibacterial agents on each bacterium in the milk microbiota may differ, and it may take some time before the effects can be perceived as changes in the microbiota composition. Bonsaglia et al. found that Firmicutes, Proteobacteria, Actinobacteria and Bacteroidetes were abundant regardless of time or treatment when healthy mammary glands in the dry period were treated with antimicrobials [14]. In addition, intramammary therapy with ceftiofur hydrochloride for mastitis with Gram-negative bacteria such as *E. coli*, *Klebsiella* spp. did not affect the relative abundance of milk microbiota and Enterobacteriaceae [28]. However, according to the results of this study, the proportion of Firmicutes in milk increased in the antimicrobial treatment groups (NT-AMT and AMT-AMT groups). Therefore, the mammary microbiota in mastitic quarters was changed via penicillin—kanamycin treatment. In contrast, the milk samples obtained in this study were obtained after milking, so it is unlikely that a large amount of accumulated dead bacteria would be present. Because Firmicutes includes more species than Actinobacteria, it is possible that the antimicrobial agents affected only a small portion of the Firmicutes, and thus did not cause a significant decrease in the amount of Firmicutes; however, the absolute numbers were not determined, so it was not possible to determine whether the overall bacterial abundance or the amount of a single bacterial species increased or decreased.

In the present study, all bovine mastitis cases were eventually clinically cured, but both the SCS and NAGase activity values on day 7 were significantly lower than on day 0 or day 1, and the microbiota composition significantly changed only in the groups treated with antimicrobials, i.e., the NT-AMT and AMT-AMT groups. These changes are thought to be due to the effective elimination of the pathogens and the resolution of the inflammation as a result of treatment with antimicrobials to which the bacteria were susceptible [29]. It has been reported that antimicrobial treatment is ineffective for mastitis caused by *E. coli*, and that it does not affect the composition of the microbiota [28]; although there have been a few studies reporting similar results, the reasons have yet to be determined. However, in the present study, we used antimicrobials containing penicillin, which is highly effective against Gram-positive bacteria [30] and may have enough effect to change milk microbiota. Interestingly, the cases in the NT-NT group were also clinically cured, although the microbiota composition did not change in this group. It Is unknown whether these cases were bacteriologically cured or not, but the inflammation subsided over time, and the cases were clinically judged to have been spontaneously cured. This may be because the causative bacterium in the NT-NT group was the CNS, where spontaneous healing has been reported [31] in many cases. However, we only observed up to 7 days, and considering the recurrence rate, we do not think that antibiotic treatment is meaningless. It is possible that the reason the milk microbiota composition did not change was because no antimicrobial agents were used. These results indicated that the milk microbiota of udders cured from mild mastitis caused by Gram-positive bacteria are not always the same. In addition, it is unclear whether the composition of the milk microbiota of the cured cases was similar to the indigenous milk microbiota composition of this herd. According to previous studies, antimicrobials may have long-term effects on the intestinal microbiota [8,32]. Therefore, because the observation period of this study was only about 1 week, it is possible that the milk microbiota composition of the cured cases had not yet returned to that of normal non-mastitic cows. This cannot be confirmed until the original healthy microbiota of this herd is determined.

The present study has several other potential limitations that should be acknowledged. Firstly, as mentioned above, the indigenous milk microbiota of the herd used in the present study has not yet been examined. In future studies, milk samples from cows without mastitis should be collected and analyzed with the same methods used in this study for comparison with the milk microbiota of the cured cases. Secondly, because the population mean, variance and standard deviation of milk microbiota in this herd were unknown before the trial, we could not calculate the appropriate sample size to use. In particular, the number of cows in the NT-NT group was small. This is because the number of non-treated cows was reduced as much as possible due to animal welfare concerns. Finally, because the meta-genome analysis was carried out using a 16S rRNA gene amplicon sequence, we could not determine the absolute number of each bacterial species. It is necessary to investigate the absolute numbers by combining other methods such as quantitative PCR [33,34].

Despite research limitations above, this study clarified how intramammary antimicrobial administration affects the milk microbiota of mastitic quarters and helps to enable a better understanding of mastitis.

## 5. Conclusions

The results of the present study provided novel evidence that the use of intramammary penicillin—kanamycin treatment for mild mastitis due to coagulase-negative staphylococci or *Streptococcus* spp. other than *S. uberis* reduces not only the SCS and NAGase activity in milk, but also changes the composition of the milk microbiota from day 2 until at least day 7. Additionally, antimicrobial treatment may lead to a milk microbiota composed of similar bacterial species.

## Figures and Tables

**Table 1:** PCR mixture (one sample) and PCR condition for amplification of V1–V9 region of the 16S rRNA gene.

PCR Mixture		PCR Condition			
Forward primer [23]	0.25 µM (Final conc.)	5′-GACGGGCGGTGWGTRCA-3′			
Reverse primer [23]	0.25 µM (Final conc.)	5′-AGRGTTYGATYMTGGCTCAG-3′			
KAPA HiFi HotStart ReadyMix	10 µL	Amplification			
		Step	Duration	Temperature	Cycles
Nuclease Free Water	7 µL	Initial denaturation	5 min	95 °C	1
*Haloarcula japonica* *	1 µL (2.8 × 10^3^ cells)	Denaturation	20 s	98 °C	35
Genomic DNA	1 µL (100 ng)	Annealing	15 s	69 °C	35
		Extension	60 s	72 °C	35
		Final extension	5 min	72 °C	1
		Hold	∞	4 °C	1

*: Spiked-in as an internal control.

**Table 2:** The use of antimicrobial agents in each treatment group and cow characteristics enrolled in this study.

Treatment Group	Antimicrobial Treatment		*n*	Characteristics		
	Day 0	Day 1		Age (Mean ± S.D.)	DIM (Mean ± S.D.)	Parity (Mean ± S.D.)
NT-NT	NO	NO	3	3.20 ± 1.25	126.67 ± 119.56	1.33 ± 0.58
NT-AMT	NO	YES	7	3.36 ± 1.29	134.29 ± 88.27	1.86 ± 1.07
AMT-AMT	YES	YES	6	3.50 ± 0.67	226.67 ± 128.13	1.83 ± 0.75
*p* value *				0.8047	0.425	0.6124

NT: no treatment; AMT: antimicrobial treatment; DIM: the days in milk. *: Kruskal–Wallis test.

**Table 3:** Bacterial isolation and antimicrobial susceptibilities of antimicrobials of each sample based on the agar disk diffusion further simplified (simplified ADD) method.

Sample No.	Treatment Group	Isolation	BBL Crystal Result	Inhibition Circle Diameter (mm)		Susceptibility	
				Penicillin	Kanamycin	Penicillin	Kanamycin
1	NT-NT	CNS	*Staphylococcus equorum*	ND	ND	ND	ND
2	NT-NT	CNS	*Staphylococcus equorum*	ND	ND	ND	ND
3	NT-NT	CNS	*Staphylococcus saprophyticus*	ND	ND	ND	ND
4	NT-AMT	OS	*Streptococcus dysgalactiae*	29.7	9.3	S	R
5	NT-AMT	CNS	*Staphylococcus haemolyticus*	38.0	31.2	S	S
6	NT-AMT	OS	*Streptococcus equinus*	28.8	7.0	S	R
7	NT-AMT	OS	*Streptococcus porcinus*	25.9	7.0	S	R
8	NT-AMT	OS	*Streptococcus bovis*	17.6	8.0	S	R
9	NT-AMT	CNS	*Staphylococcus intermedius*	26.1	7.0	S	R
10	NT-AMT	CNS	*Staphylococcus sciuri*	28.6	25.1	S	S
11	AMT-AMT	OS	*Streptococcus dysgalactiae*	32.1	10.0	S	R
12	AMT-AMT	OS	*Streptococcus dysgalactiae*	29.3	9.6	S	R
13	AMT-AMT	OS	*Streptococcus dysgalactiae*	29.6	10.3	S	R
14	AMT-AMT	OS	*Streptococcus vestibularis*	27.6	7.0	S	R
15	AMT-AMT	OS	*Streptococcus bovis*	24.9	8.0	S	R
16	AMT-AMT	OS	*Streptococcus dysgalactiae*	29.0	10.6	S	R

NT: no treatment; AMT: antimicrobial treatment; CNS: coagulase negative staphylococci; OS: *Streptococcus* spp. other than *Streptococcus uberis*, ND: not done; S: Sensitive; R: Resistant.

## Data Availability

Not applicable.

## References

[1] Shinozuka Y. (2019). Consider antibiotics treatment for non-severe clinical mastitis in dairy cattle. J. Farm Anim. Infect. Dis..

[2] Oliveira L., Hulland C., Ruegg P. (2013). Characterization of clinical mastitis occurring in cows on 50 large dairy herds in Wisconsin. J. Dairy Sci..

[3] Fernández L., Langa S., Martin V., Jiménez E., Martín R., Rodríguez J. (2013). The microbiota of human milk in healthy women. Cell. Mol. Biol..

[4] Yeoman C.J., Ishaq S.L., Bichi E., Olivo S.K., Lowe J., Aldridge B.M. (2018). Biogeographical differences in the infuence of maternal microbial sources on the early successional development of the bovine neonatal gastrointestinal tract. Sci. Rep..

[5] Falentin H., Rault L., Nicolas A., Bouchard D.S., Lassalas J., Lamberton P., Aubry J.-M., Marnet P.-G., Le Loir Y., Even S. (2016). Bovine Teat Microbiome Analysis Revealed Reduced Alpha Diversity and Significant Changes in Taxonomic Profiles in Quarters with a History of Mastitis. Front. Microbiol..

[6] Oikonomou G., Bicalho M.L., Meira E., Rossi R.E., Foditsch C., Machado V.S., Teixeira A.G.V., Santisteban C., Schukken Y.H., Bicalho R.C. (2014). Microbiota of cow’s milk; distinguishing healthy, sub-clinically and clinically diseased quarters. PLoS ONE.

[7] Patangia D.V., Ryan C.A., Dempsey E., Ross R.P., Stanton C. (2021). Impact of antibiotics on the human microbiome and consequences for host health. MicrobiologyOpen.

[8] Ramirez J., Guarner F., Fernandez L.B., Maruy A., Sdepanian V.L., Cohen H. (2020). Antibiotics as major disruptors of gut microbiota. Front. Cell. Infect. Microbiol..

[9] Ji S., Jiang T., Yan H., Guo C., Liu J., Su H., Algongo G.M., Shi H., Wang Y., Cao Z. (2018). Ecological restoration of antibiotic-disturbed gastrointestinal microbiota in foregut and hindgut of cows. Front. Cell. Infect. Microbiol..

[10] Yousif M.H., Li J., Li Z., Alugongo G.M., Ji S., Li Y., Wang Y., Li S., Cao Z. (2018). Low concentration of antibiotics modulates gut microbiota at different levels in pre-weaning dairy calves. Microorganisms.

[11] Soto A., Martın V., Jimenez E., Mader I., Rodrıguez J.M., Fernandez L. (2014). Lactobacilli and *Bifidobacteria* in human breast milk: Influence of antibiotherapy and other host and clinical factors. J. Pediatr. Gastroenterol. Nutr..

[12] Hermansson H., Kumar H., Collado M.C., Salminen S., Isolauri E., Rautava S. (2019). Breast Milk Microbiota Is Shaped by Mode of Delivery and Intrapartum Antibiotic Exposure. Front. Nutr..

[13] Basbas C., Aly S., Okello E., Karle B.M., Lehenbauer T., Williams D., Ganda E., Wiedmann M., Pereira R.V. (2022). Effect of Intramammary Dry Cow Antimicrobial Treatment on Fresh Cow’s Milk Microbiota in California Commercial Dairies. Antibiotics.

[14] Bonsaglia E.C.R., Gomes M.S., Canisso I.F., Zhou Z., Lima S.F., Rall V.L.M., Oikonomou G., Bicalho R.C., Lima F.S. (2017). Milk microbiome and bacterial load following dry cow therapy without antibiotics in dairy cows with healthy mammary gland. Sci. Rep..

[15] Roberson J.R. (2003). Establishing treatment protocols for clinical mastitis. Veter Clin. N. Am. Food Anim. Pract..

[16] Dahlberg J., Williams J., McGuire M., Peterson H., Östensson K., Agenäs S., Dicksved J., Waller K.P. (2020). Microbiota of bovine milk, teat skin, and teat canal: Similarity and variation due to sampling technique and milk fraction. J. Dairy Sci..

[17] Kurumisawa T., Kawai K., Shinozuka Y. (2021). Verification of a simplified disk diffusion method for antimicrobial susceptibility testing of bovine mastitis isolates. Jpn. J. Vet. Res..

[18] Kawai K., Hayashi T., Kiku Y., Chiba T., Nagahata H., Higuchi H., Obayashi T., Itoh S., Onda K., Arai S. (2013). Reliability in somatic cell count measurement of clinical mastitis milk using DeLaval cell counter. Anim. Sci. J..

[19] Wiggans G.R., Shook G.E. (1987). A Lactation Measure of Somatic Cell Count. J. Dairy Sci..

[20] De Coster W., D’Hert S., Schultz D.T., Cruts M., Van Broeckhoven C. (2018). NanoPack: Visualizing and processing long-read sequencing data. Bioinformatics.

[21] GenomeSync. http://genomesync.org.

[22] Federhen S. (2012). The NCBI Taxonomy database. Nucleic Acids Res..

[23] Klindworth A., Pruesse E., Schweer T., Peplies J., Quast C., Horn M., Glöckner F.O. (2013). Evaluation of General 16S Ribosomal RNA Gene PCR Primers for Classical and Next-Generation Sequencing-Based Diversity Studies. Nucleic Acids Res..

[24] Kanda Y. (2013). Investigation of the freely available easy-to-use software ‘EZR’ for medical statistics. Bone Marrow Transplant..

[25] Clarke K.R. (1993). Non-parametric multivariate analyses of changes in community structure. Aust. J. Ecol..

[26] Hammer Ø., Harper D.A.T., Ryan P.D. (2001). PAST: Paleontological statistics software package for education and data analysis. Palaeontol. Electron..

[27] Segata N., Izard J., Waldron L., Gevers D., Miropolsky L., Garrett W.S., Huttenhower C. (2011). Metagenomic biomarker discovery and explanation. Genome Biol..

[28] Ganda E.K., Bisinotto R.S., Lima S.F., Kronauer K., Decter D.H., Oikonomou G., Schukken Y.H., Bicalho R.C. (2016). Longitudinal metagenomic profiling of bovine milk to assess the impact of intramammary treatment using a third-generation cephalosporin. Sci. Rep..

[29] Pyörälä S. (2003). Indicators of inflammation in the diagnosis of mastitis. Veter. Res..

[30] Kalmus P., Simojoki H., Orro T., Taponen S., Mustonen K., Holopainen J., Pyörälä S. (2014). Efficacy of 5-day parenteral versus intramammary benzylpenicillin for treatment of clinical mastitis caused by gram-positive bacteria susceptible to penicillin in vitro. J. Dairy Sci..

[31] Taponen S., Pyörälä S. (2009). Coagulase-negative staphylococci as cause of bovine mastitis -not so different from?. Vet. Microbiol..

[32] Jernberg C., Löfmark S., Edlund C., Jansson J.K. (2010). Long-term impacts of antibiotic exposure on the human intestinal microbiota. Microbiology.

[33] Jian C., Luukkonen P., Yki-Järvinen H., Salonen A., Korpela K. (2020). Quantitative PCR provides a simple and accessible method for quantitative microbiota profiling. PLoS ONE.

[34] Matsuki T., Watanabe K., Fujimoto J., Takada T., Tanaka R. (2004). Use of 16S rRNA Gene-Targeted Group-Specific Primers for Real-Time PCR Analysis of Predominant Bacteria in Human Feces. Appl. Environ. Microbiol..

